# Pore structure and surface area of silica SBA-15: influence of washing and scale-up

**DOI:** 10.3762/bjnano.2.13

**Published:** 2011-02-16

**Authors:** Jörg P Thielemann, Frank Girgsdies, Robert Schlögl, Christian Hess

**Affiliations:** 1 Abteilung Anorganische Chemie, Fritz-Haber-Institut der Max-Planck-Gesellschaft, Faradayweg 4–6, 14195 Berlin, Germany; 2 Eduard-Zintl-Institut für Anorganische und Physikalische Chemie, Technische Universität Darmstadt, Petersenstr. 20, 64287 Darmstadt, Germany

**Keywords:** SBA-15, scale-up, silica mesoporous material, tensile strength effect, washing

## Abstract

The removal of the surfactant (EO_20_PO_70_EO_20_) by washing before final calcination is a critical step in the synthesis of silica SBA-15. In contrast to washing with pure water or ethanol, washing with water *and* ethanol may, depending on the quantity of solvent used, alter the homogeneity and order of the pores, but also lead to an increase of the surface area of SBA-15. A reduction of solvent volume and a controlled washing protocol allow the synthesis of high surface area SBA-15 materials with a narrow monomodal pore size distribution. For larger batch sizes the influence of the quantity of solvent on the quality of the SBA-15 is reduced.

## Introduction

SBA-15 is a mesoporous silica sieve based on uniform hexagonal pores with a narrow pore size distribution and a tunable pore diameter of between 5 and 15 nm [1]. The thickness of the framework walls is about 3.1 to 6.4 nm, which gives the material a higher hydrothermal and mechanical stability than, for instance, MCM-41 [2]. The high internal surface area of typically 400–900 m^2^/g makes SBA-15 a well suited material for various applications. It can be used in environmental analytics for adsorption and separation [3–4], advanced optics [5–6], as a support material for catalysts [7–8] and as a template for the production of nanostructured carbon or platinum replica [9–10].

SBA-15 is synthesized in a cooperative self-assembly process under acidic conditions using the triblock copolymer Pluronic 123 (EO_20_PO_70_EO_20_) as template and tetraethoxysilane (TEOS) as the silica source [11]. After synthesis, the template can be removed by calcination [1,12], washing [13–14], reflux extraction [1,12], acid [15], H_2_O_2_ treatment [16], extraction with supercritical CO_2_ [17] and microwave digestion [18]. In the literature template removal is often carried out using pure solvents such as water [19], acetone [7] or ethanol [20–21]. According to Bae et al. using ethanol instead of water is three times more effective in removing the template from the SBA-15 framework, although the Pluronic 123 cannot be removed completely from the SBA-15 by washing, as shown by thermogravimetric analysis (TGA) [22]. Also the more effective template removal by ethanol is connected with shrinkage of the SBA-15 structure as observed by Ko et al. [13].

To the best of our knowledge there are no reports on SBA-15 synthesis using a washing approach with two solvents. In the following we will show that the use of two solvents while washing can lead to an increase of the surface area and that for small batches the volume of the solvents has an impact on the surface area and pore size distribution of SBA-15. Also the influence of scaling up to 27 g per batch on the properties of SBA-15 is discussed. Up until now the largest synthesis scale as reported by Tkachenko et al. corresponded to approximately 24.5 g [23].

The paper is organised as follows: In the first section the influence of washing freshly synthesised SBA-15 with water or ethanol is discussed. The second section deals with a combined washing approach using water and ethanol. Particular emphasis is put on the effect of the solvent quantities being used while washing. The third section addresses the issue of scaling up the SBA-15 synthesis to 9 times the size described in the original procedure by Zhao et al. [1,12].

## Results and Discussion

### Template removal by washing with a single solvent

The removal of the Pluronic 123 template (EO_20_PO_70_EO_20_) by washing prior to calcinations is a crucial step in the synthesis of SBA-15. Therefore, a series of experiments was conducted to understand first the effect of each solvent separately (water, ethanol), and then the effect of a combination of these two solvents while washing. To investigate the batch size dependence while washing with a single solvent, a 9× batch was separated into two 1× half batches and two 3× half batches (see Experimental section for detailed information). As the results for both half batches were similar, only the 1× half batch will be discussed in the following.

**Figure 1:**
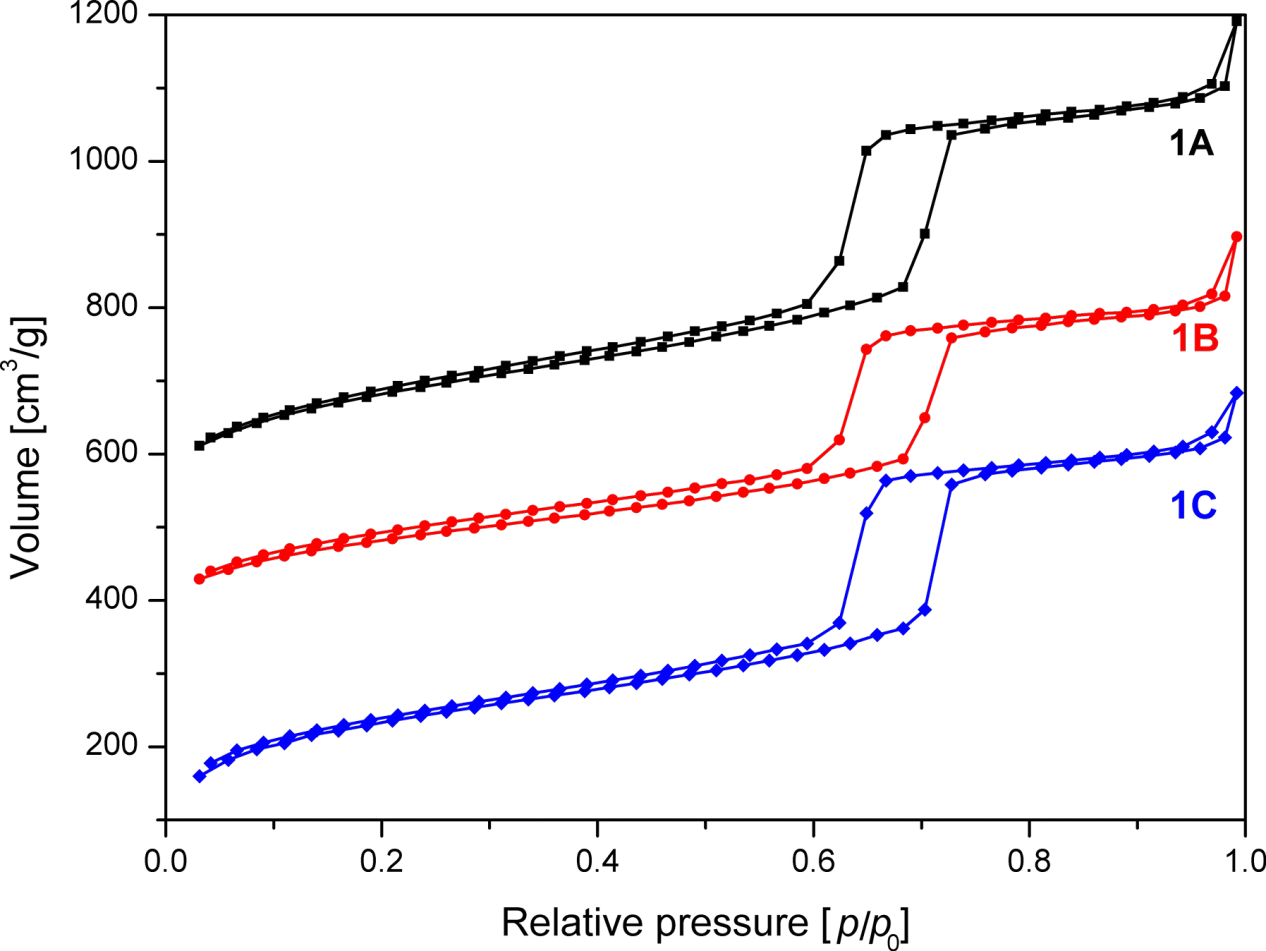
N_2_ adsorption/desorption isotherms of SBA-15 unwashed (**1A**), after washing with 30 mL ethanol (**1B**) and 30 mL water (**1C**). The isotherms are offset by 450 (**1A**) and 300 (**1B**) cm^3^/g.

The shape of the isotherms of sample **1** (Figure 1) is almost ideally type-IV and no change of the hysteresis was observed with the different washing procedures. This means that the homogeneity and order of the hexagonal pores were not altered by washing. The surface area and the pore volume of the untreated reference sample **1A** and the water treated sample **1C** is nearly the same, whereas the surface area and pore volume of the ethanol treated sample **1B** is significantly reduced by 150 m^2^/g and 0.2 cm^3^/g. The shrinking of pore volume and surface area while washing with pure ethanol seems to be due to more efficient removal of the surfactant from the meso- and micropores than with pure water [13]. The shrinking also affected the ratio between micro- and mesopores. The ethanol washed sample **1B** exhibited more and the water washed sample **1C** less micropore volume than the unwashed reference samples **1A** (Table 1).

**Table 1:** Surface and porosity characteristics of SBA-15 samples washed with pure water, ethanol and a combination of ethanol and water.

SBA-15 sample	washing procedure^a^	*S*_Micro_ (m^2^/g)^b^	*S*_Total_ (m^2^/g)^c^	*S*_Micro_ /*S*_Total_^d^	*D*_P_ (Å)^e^	*a*_0_ (Å)^f^	*a*_0_−*D*_P_ (Å)^g^	*V*_Total_ (cm^3^/g)^h^

**1A**	no washing	263	700	0.38	70	108	38	0.98
**1B**	30 mL ethanol	300	551	0.54	73	110	37	0.78
**1C**	30 mL water	213	709	0.30	70	108	38	0.94
**2A**	no washing	267	671	0.40	68	107	39	0.88
**2B**	5 mL ethanol/water	381	817	0.47	70	109	39	1.01
**2C**	30 mL ethanol/water	215	573	0.38	61	100	39	0.62
**3A**	no washing	297	758	0.39	70	111	41	0.88
**3B**	5 mL ethanol/water (1× half batch)	389	872	0.45	70	107	37	1.11
**3C**	35 mL ethanol/water (1× half batch)	343	755	0.45	68	109	41	0.87
**3D**	120 mL ethanol/water (3× half batch)	354	790	0.45	70	109	39	0.96
**3E**	350 mL ethanol/water (9× half batch)	364	838	0.43	70	111	41	1.06

^a^solvent volume used per washing cycle; ^b^micropore surface area; ^c^total BET surface area; ^d^fraction of the micropore surface area of the total BET surface area; ^e^pore diameter determined from the adsorption isotherms by the NLDFT method; ^f^unit-cell parameter (*a*_0_) determined from small-angle XRD; ^g^pore wall thickness estimated by subtracting the pore diameter value (*D*_P_) from the hexagonal unit-cell dimension (*a*_0_); ^h^total pore volume.

**Figure 2:**
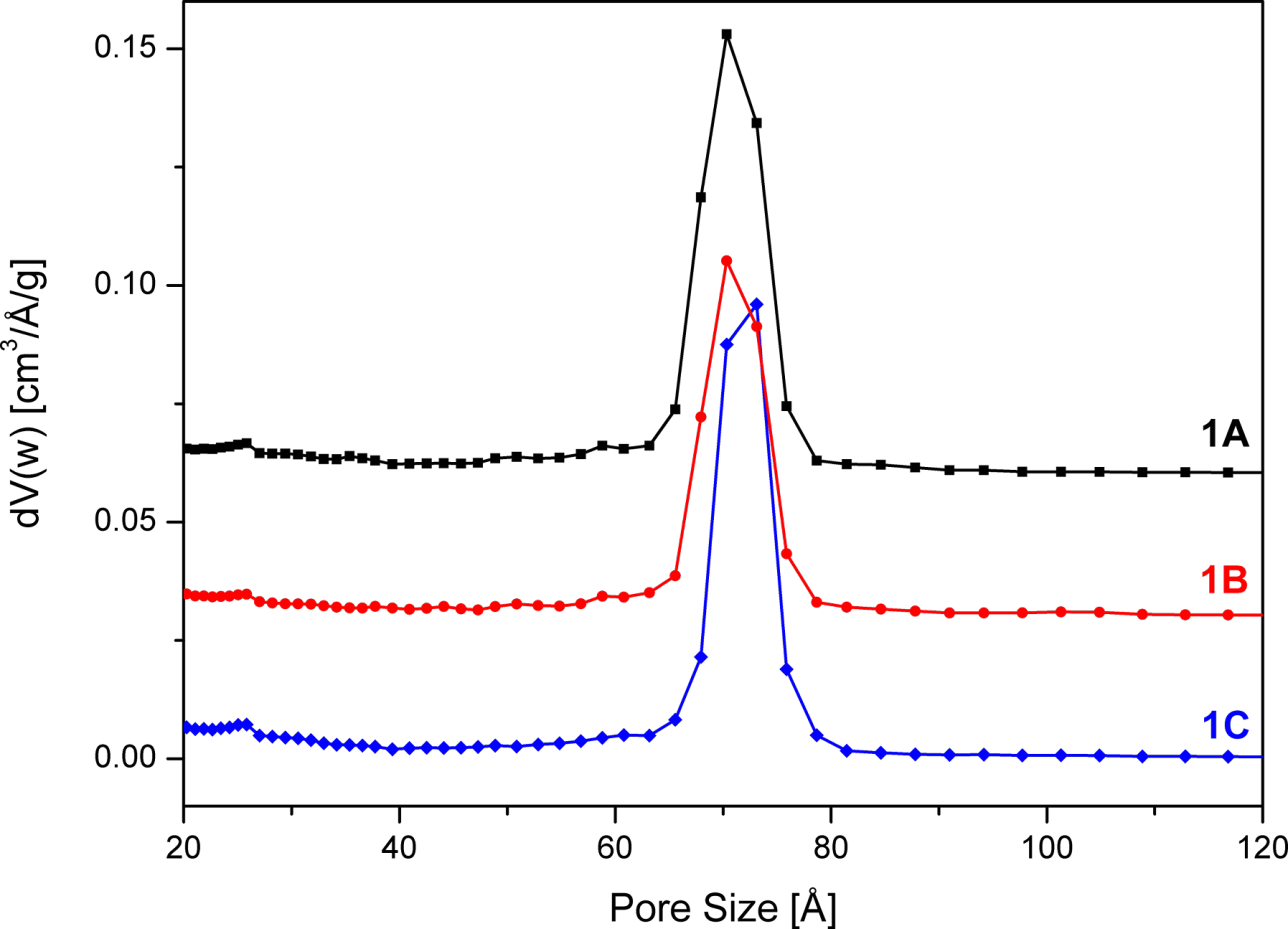
NLDFT pore size distributions of SBA-15 unwashed (**1A**), after washing with 30 mL ethanol (**1B**) and 30 mL water (**1C**) calculated from the adsorption branch of the isotherm. The pore size distributions are offset by 0.06 (**1A**) and 0.03 (**1B**) cm^3^/Å/g.

**Figure 3:**
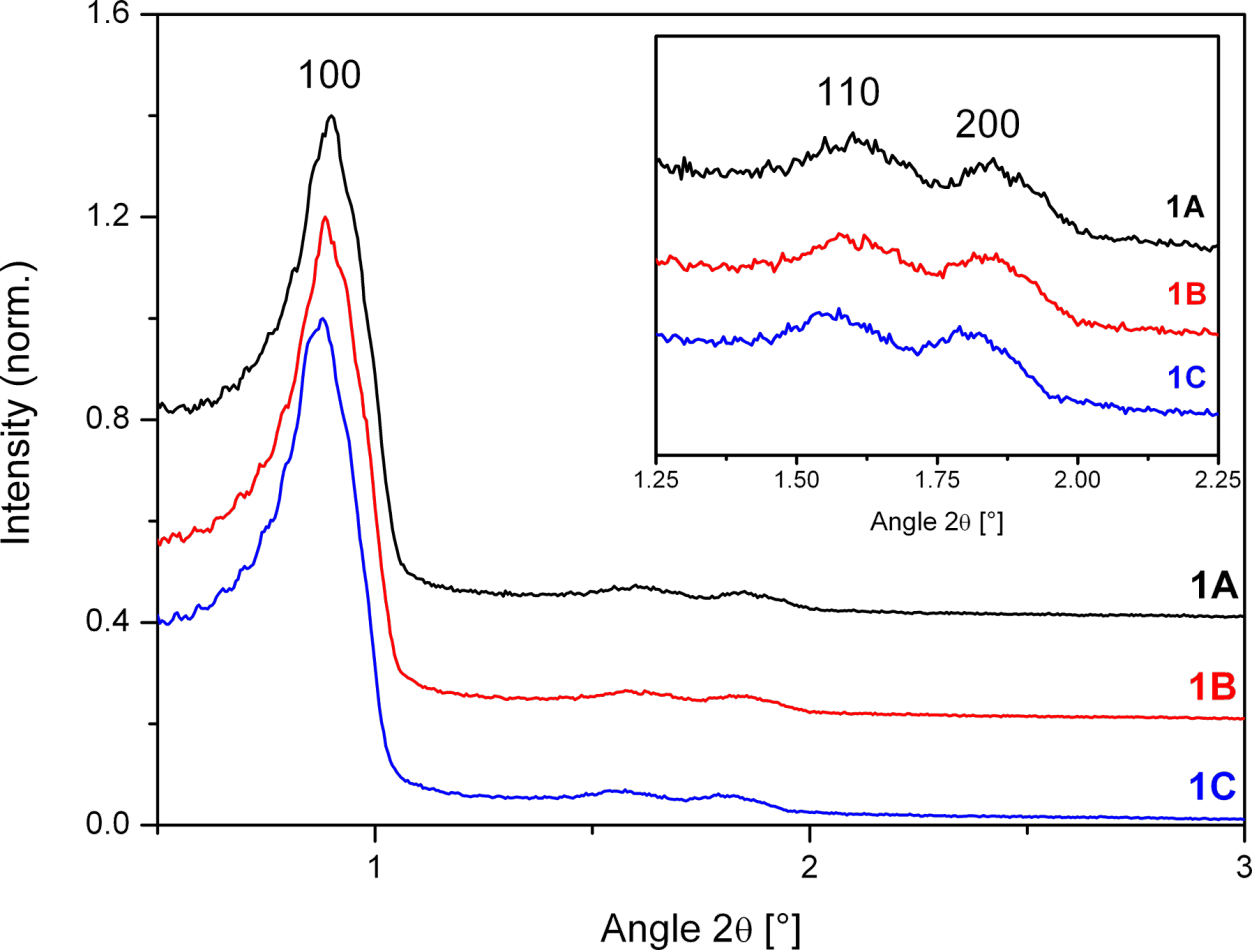
Small angle XRD of SBA-15 unwashed (**1A**), after washing with 30 mL ethanol (**1B**) and 30 mL water (**1C**). The intensities are normalized to the (100) reflection and offset by 0.4 (**1A**) and 0.2 (**1B**).

Figure 2 depicts the pore size distributions of the adsorption branch of the isotherm for the three samples, which are quite similar. The pore size distributions were characterized by a maximum at 70 Å (samples **1A** and **1C**) and 73 Å (sample **1B**) and a FWHM of 8 Å was observed. The high order of the mesopores of samples **1A**–**C** – compared to SBA-15 samples **2C** and **3C** reported in later sections – was also corroborated by XRD results (Figure 3). Independent of the washing procedure the (100), (110) and (200) reflections occur at almost the same position with the same relative intensity.

### Influence of combined washing with ethanol and water

**Figure 4:**
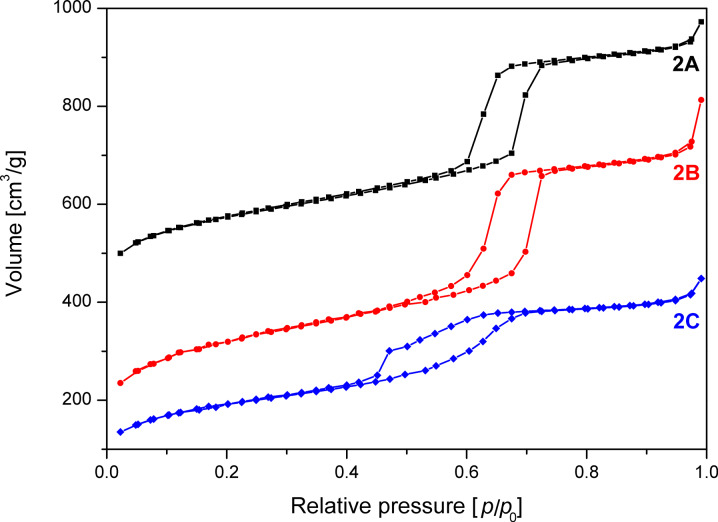
N_2_ adsorption/desorption isotherms of SBA-15 unwashed (**2A**), after washing with 5 mL (**2B**) and 30 mL (**2C**) quantities of solvent. The isotherms are offset by 350 (**2A**) and 50 (**2B**) cm^3^/g.

In contrast to washing with a pure solvent, the combined washing with ethanol and water may lead to an increase of surface area (Table 1), but also damages the material. The damage is observed as a bulge in the desorption branch of the BET isotherm. To investigate the possible sources for this decreased homogeneity of the pores the following potential influences on the synthesis were examined and excluded: (i) temperature in the first synthesis step a) by precise temperature stabilization at 35 °C and avoidance of any temperature fluctuations b) by increasing the synthesis temperature to 37 °C, (ii) variation of the addition velocity of tetraethoxysilane (TEOS), (iii) stirring velocity during the synthesis and TEOS addition, (iv) heating rate and temperature fluctuation at the aging step (85 °C), (v) influence of the cooling rate after aging, (vi) tightness of the bottle while aging and (vii) influence of grinding after synthesis. Van der Voort et al. and Kruk et al. [24–25] report that an increased TEOS/surfactant ratio can influence the structure of the SBA-15. Therefore, according to their recommendation (viii) the TEOS/surfactant ratio was fixed to 58:1 for all samples to exclude it as source for the observed disorder. Importantly, it was found that under the chosen conditions only the amount of solvent used in the washing process had a significant influence on the shape of the isotherm of the final product (Figure 4).

**Figure 5:**
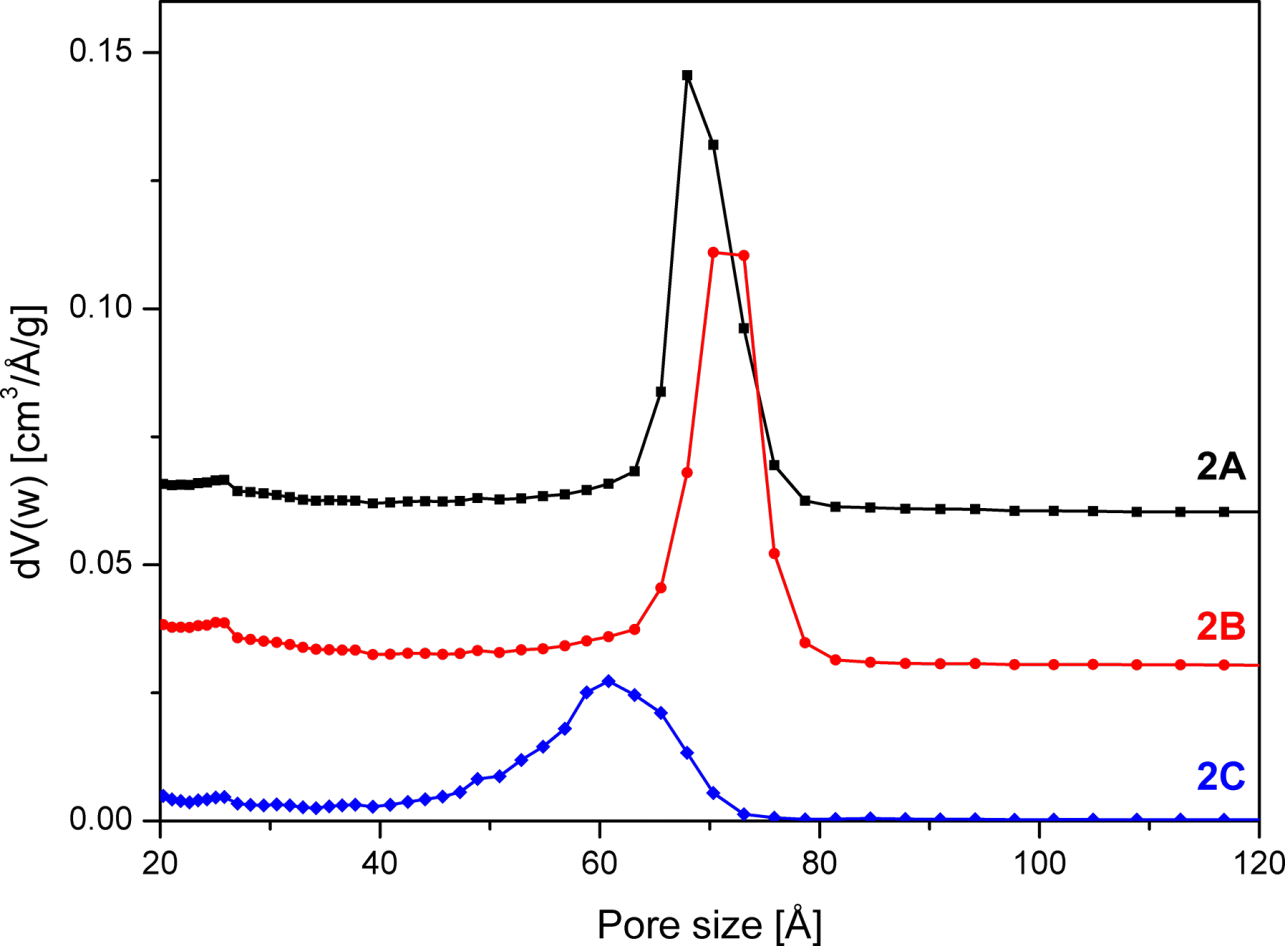
NLDFT pore size distribution of SBA-15 unwashed (**2A**), after washing with 5 mL (**2B**) and 30 mL (**2C**) quantities of solvent calculated from the adsorption branch of the isotherm. The pore size distributions are offset by 0.05 (**2A**) and 0.025 (**2B**) cm^3^/Å/g.

**Figure 6:**
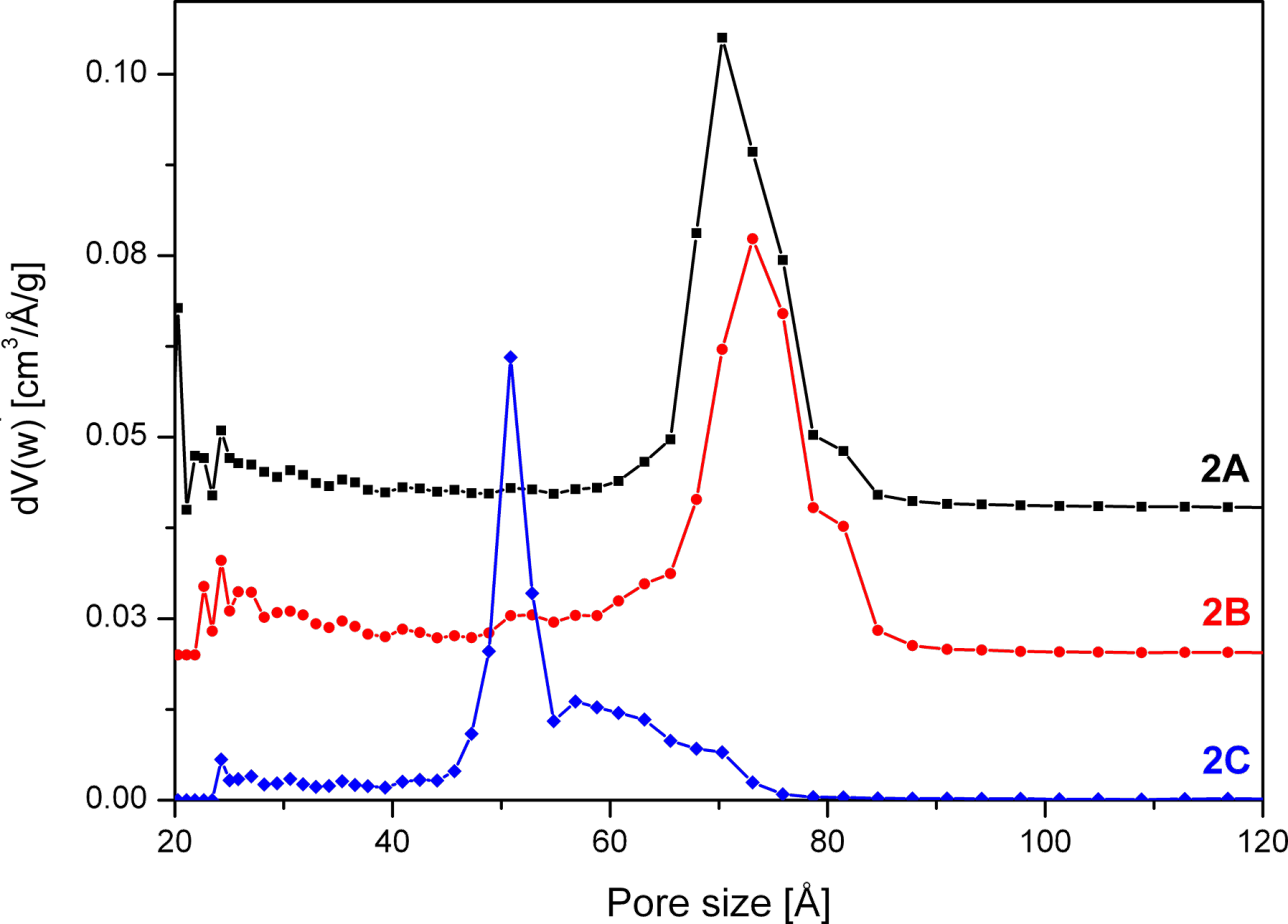
NLDFT pore size distribution of SBA-15 unwashed (**2A**), after washing with 5 mL (**2B**) and 30 mL (**2C**) quantities of solvent calculated from the desorption branch of the isotherm. The pore size distributions are offset by 0.5 (**2A**) and 0.25 (**2B**) cm^3^/Å/g.

To gain insight into the influence of solvent volume, a single batch (sample **2**) was split into two half batches. A reference sample **2A** of 80 mg, which was not washed, was taken before splitting into two half batches. One of the half batches was gently washed with 5 mL (**2B**) and the other with 30 mL (**2C**) of solvent. All samples were subsequently calcined at 550 °C. The resulting samples were analyzed regarding BET surface area, pore size distribution and pore volume (Figure 5, Figure 6 and Table 1).

The reference sample **2A**, which was not washed shows the best isotherm hysteresis and a narrow pore size distribution (FWHM = 9 Å) as calculated from the adsorption branch of the isotherm (Figure 5 and Table 1). In comparison to the other two samples, the gently washed sample **2B** shows a significantly increased surface area and a narrow pore size distribution (FWHM = 9 Å) with a maximum at 70 Å. In contrast, sample **2C** washed with plenty of solvent shows a significantly changed shape of the isotherm as evidenced by the bulge in the desorption branch at *p*/*p*_0_ = 0.45 as well as by a reduced surface area compared to sample **2B**.

The closure of the hysteresis loop at *p*/*p*_0_ values between 0.4 and 0.45 for sample **2C** can be explained by the tensile strength effect [26–27]. The effect occurs when interconnected pores filled with N_2_ at 77 K are emptied through smaller pores or narrower sections along the pore. In those pores with a diameter below 50 Å the N_2_ evaporation is delayed until a critical pressure (*p*/*p*_0_)_TSE_ is reached, at which the hemispherical meniscus collapses and the pores are immediately emptied. This also leads to the observed forced closure of the hysteresis loop as pores with smaller diameter do not show a hysteresis [28]. As a result there is a typical sharp peak at 50 Å in the pore size distribution (Figure 6), which can be considered an artefact [26]. Therefore, as recommended [27,29] the unaffected adsorption branch of the isotherm was used to calculate the pore size distribution.

Nevertheless, the pore size distribution of sample **2C** calculated from the adsorption branch of the isotherm is relatively broad and shifted to 61 Å as compared to samples **2A** and **2B**. This behaviour shows that the order of the pores in sample **2C** has decreased due to washing with increased solvent quantities. The ratio of the microporous to mesoporous surface area for **2C** stays almost constant compared to **2A**, whereas the contents of micropore surface area is increased by 7% for **2B** (Table 1).

**Figure 7:**
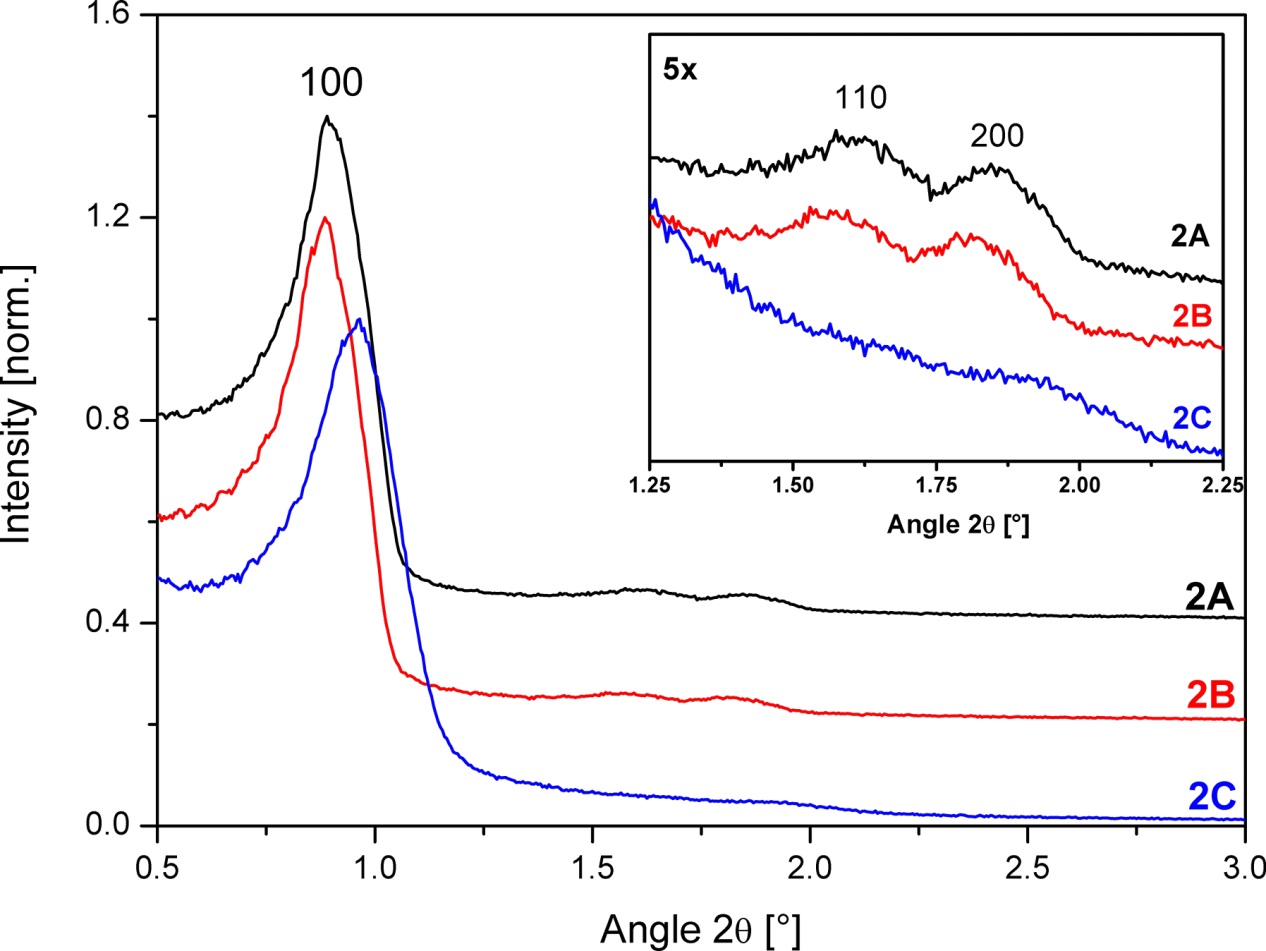
Small angle XRD of SBA-15 unwashed (**2A**), after washing with 5 mL (**2B**) and 30 mL (**2C**) quantities of solvent. The intensities are normalized to the (100) reflection and offset by 0.4 (**2A**) and 0.2 (**2B**).

The reduced order of homogeneity of the pores can be also observed in the XRD data (Figure 7), as the (110) and (200) reflections almost disappear for the extensively washed SBA-15 sample **2C**. Also the lattice constant *a*_0_ (Table 1) of sample **2C** is reduced by 9 Å compared to sample **2B**, showing that plenty of washing leads to a shrinking of the SBA-15 structure.

### Influence of scaling up on the washing effect

In the previous section it was pointed out that the amount of solvent used while washing has a significant influence on the shape of the isotherm and thus also on the pore size distribution of the resulting SBA-15. To investigate to which extent a synthesis scale up is influenced by this “washing effect” a 9× batch was split into sub factions resembling 9×, 3×, 1× half size batches. Before washing a reference sample (80 mg) was taken from the 9× batch and calcined at 550 °C. The half size batches were then washed with linearly scaled up amounts of solvent based on material weight and also calcined at 550 °C.

**Figure 8:**
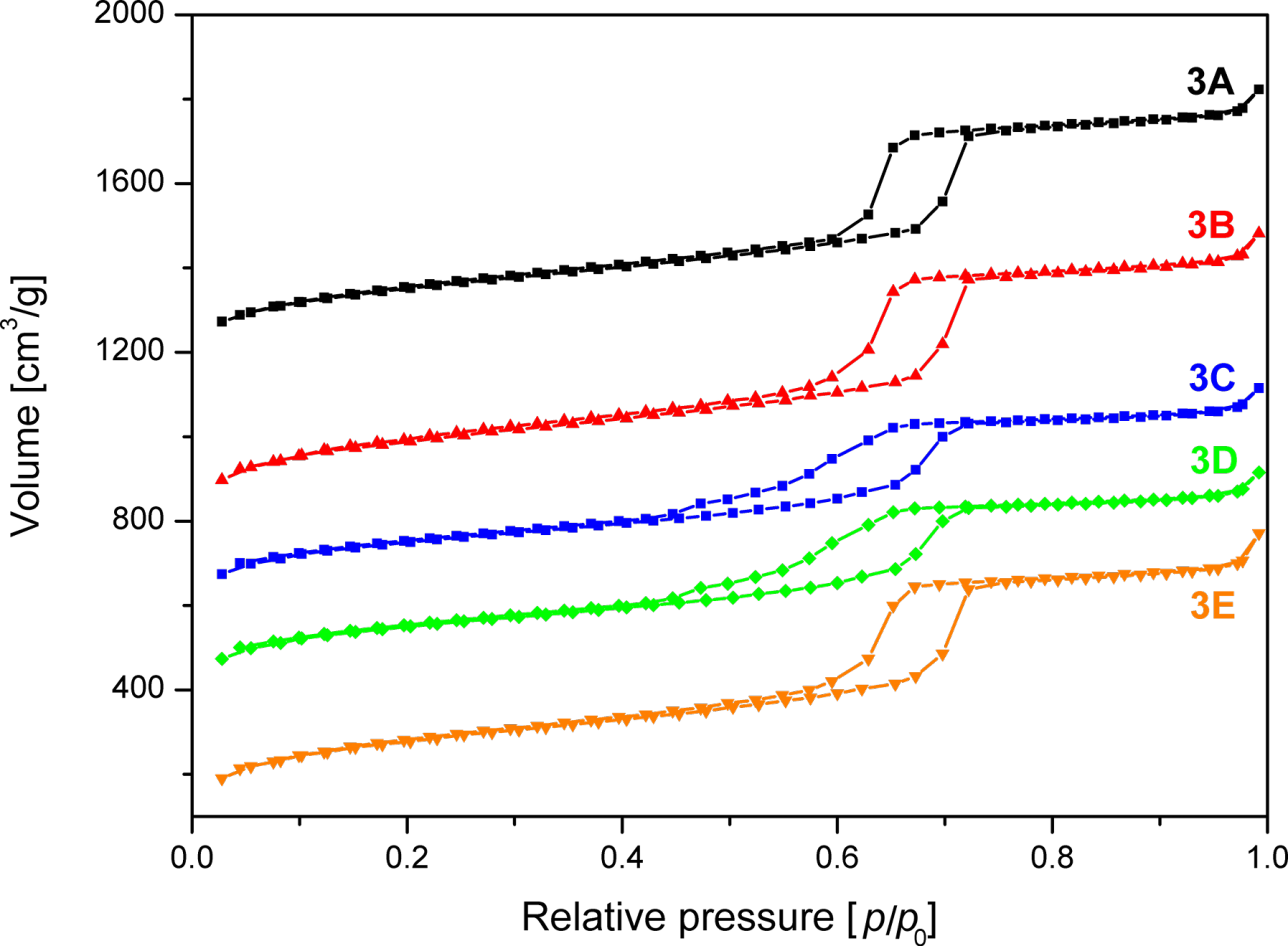
N_2_ adsorption/desorption isotherms of SBA-15 sample **3** devided into a 1× half batch unwashed (**3A**), after washing with 5 mL (**3B**) and 30 mL (**3C**), a 3× half batch washed with 120 mL (**3D**) and a 9× half batch washed with 350 mL (**3E**) quantities of solvent. The isotherms are offset by 1100 (**3A**), 700 (**3B**), 500 (**3C**), 300 (**3D**) cm^3^/g.

**Figure 9:**
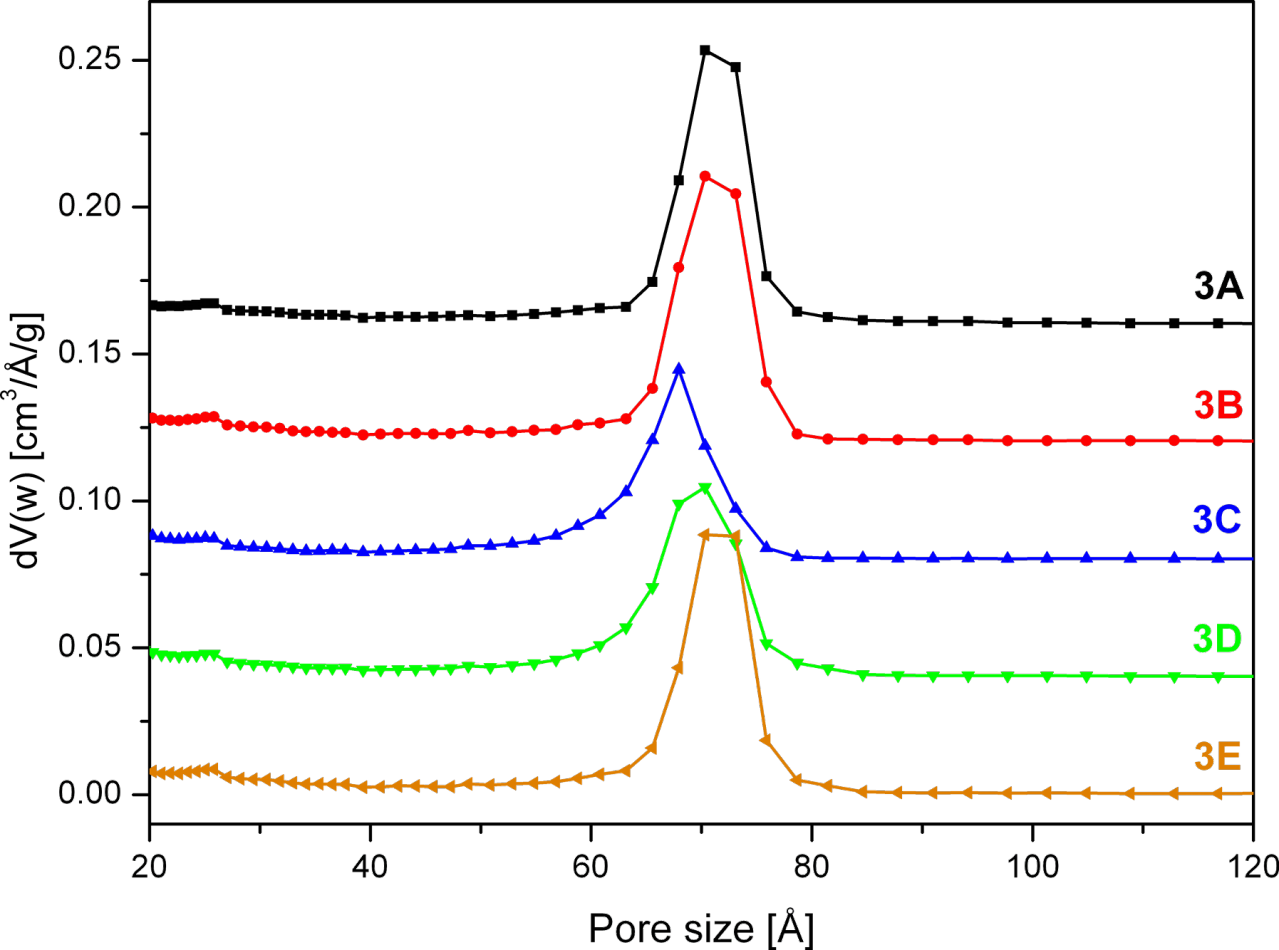
NLDFT pore size distribution of SBA-15 sample **3** calculated from the adsorption branch of the isotherm. The sample was devided into a 1× half batch unwashed (**3A**), after washing with 5 mL (**3B**) and 30 mL (**3C**), a 3× half batch washed with 120 mL (**3D**) and a 9× half batch washed with 350 mL (**3E**) quantities of solvent. The pore size distributions are offset by 0.16 (**3A**), 0.12 (**3B**), 0.08 (**3C**) and 0.04 (**3D**) cm^3^/Å/g.

Interestingly, the BET isotherms of the 1× half batch (Figure 8), which was obtained by dividing the 9× batch into smaller factions, shows similar features to the 1× half batch discussed in the previous section. The surface area was maximized in the little washed sample **3B** (Table 1) and the typical bulge in the desorption branch due to the tensile strength effect was observed at *p*/*p*_0_ = 0.45 for **3C** (Figure 8). As the extent of the bulge is lower compared to sample **2C,** the order of the mesopores appears to be slightly higher for the scaled up sample after washing with plenty of solvent. This is also reflected in the pore size distributions shown in Figure 9 as the maximum for **3C** is shifted only by 2 Å as compared to the 7 Å shift in case of **2C**. Furthermore, the peak broadening of **3C** is much lower as in case of **2C**.

Comparison of the desorption branches of the isotherms of the extensively washed samples **3C**, **3D**, **3E** (Figure 8) revealed that with increasing batch size the bulge at relative pressure 0.45 decreases and the surface area increases. This indicates that the mesopores in the material become more ordered and homogeneous during the scale-up.

**Figure 10:**
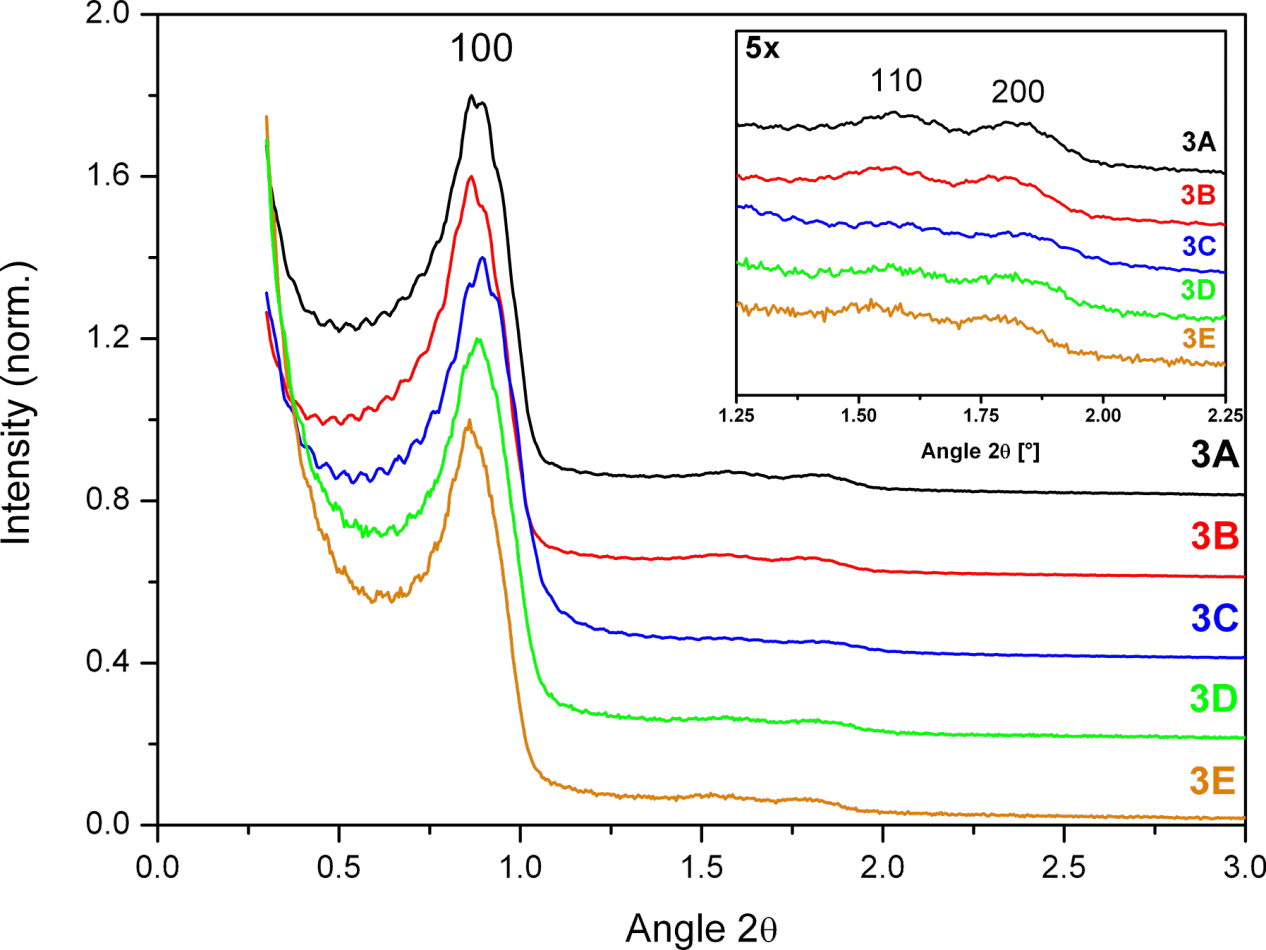
XRD of SBA-15 sample **3** devided into a 1× half batch unwashed (**3A**), after washing with 5 mL (**3B**) and 30 mL (**3C**), a 3× half batch washed with 120 mL (**3D**) and a 9× half batch washed with 350 mL (**3E**) quantities of solvent. The intesities are normalized to the (100) reflection and offset by 0.8 (**3A**), 0.6 (**3B**), 0.4 (**3C**) and 0.2 (**3D**). Note: Oscillations are an artefact in the measurement.

These results were also corroborated by small angle XRD. On the one hand, as can be seen in Figure 10, the relative intensity of the (110) and (200) reflections decreases compared to the (100) reflection from **3A, 3B** to **3C**, which shows that order of the mesopores decreases when the amount of solvent for the downscaled 1× half batch is increased. On the other hand, an increase of the mesopore order with batch size is observed for the extensively washed samples **3C**, **3D**, **3E** as the relative intensity of the (110) and (200) reflections increases.

An explanation for the higher quality of the SBA-15 may be that the washing process becomes less effective on linear scale up. This behaviour is also consistent with the observation, that for smaller batches (samples **2A**–**C** and **3A**–**C**) washing with less solvent leads to a higher quality of the SBA-15.

**Figure 11:**
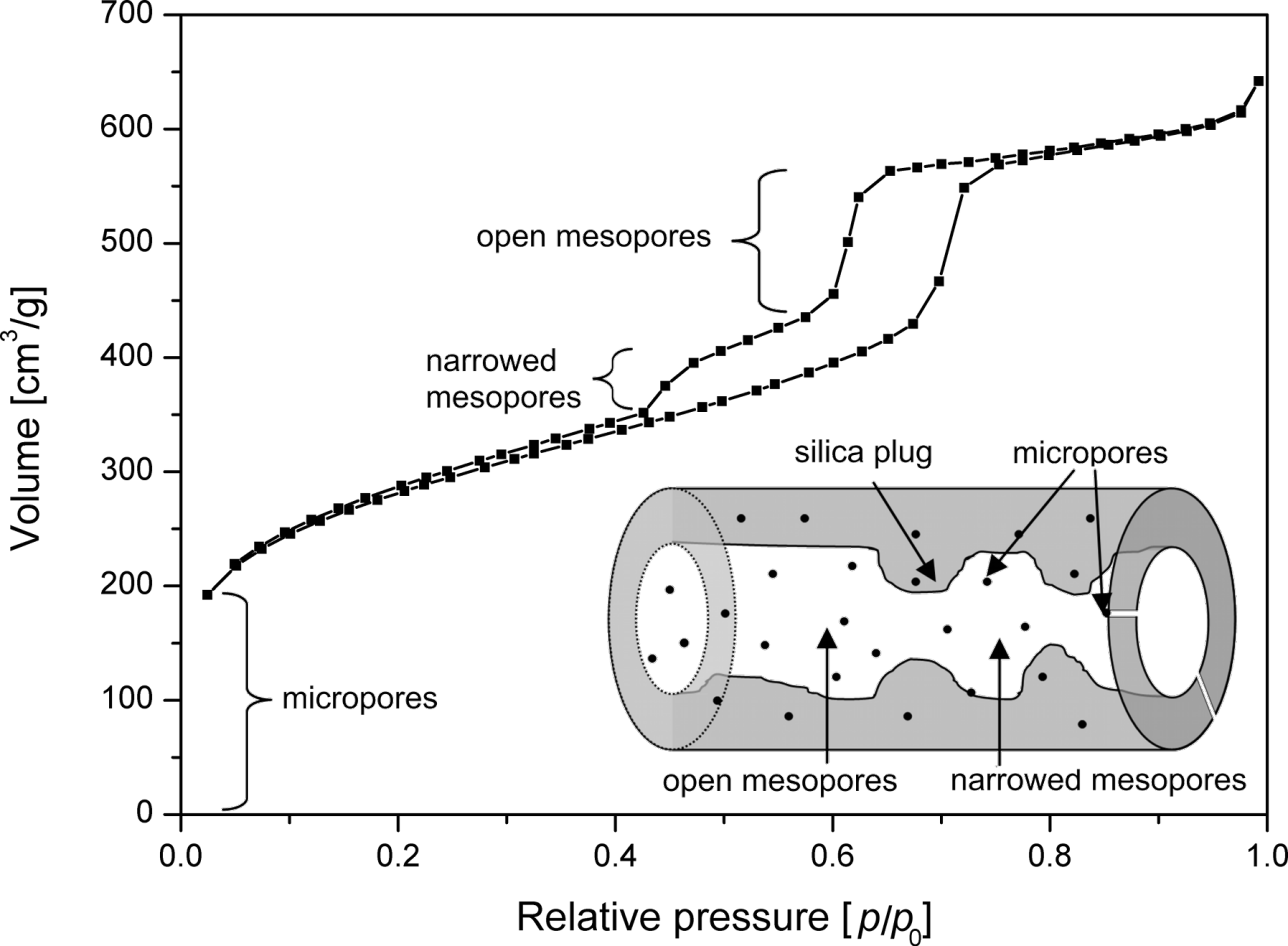
Effect of narrowed pores in the SBA-15 structure on the isotherm shape.

As described above, combined washing with ethanol and water may modify the SBA-15 mesopores. While small amounts of solvent lead to an increase in surface area without significant effects on pore structure, extensive washing strongly reduces the surface area as well as the order of the pores. A possible explanation for this behaviour could be that the template has a higher solubility in ethanol than in water. Therefore ethanol leads to a more efficient removal of the template as described by Ko and Bae [13,22]. The cleaned surface of the SBA-15 can then come into full contact with the water which being more polar is a better source for H^+^ and OH^−^ and might induce hydrolysis reactions. As a result, narrowing and widening of part of the SBA-15 mesopores may take place (Figure 11). Besides, during extensive washing the formation of blocked pores cannot be ruled out which would offer a straightforward explanation for the significant reduction in surface area. The presence of narrowed and blocked mesopores causes delayed evaporation leading to a lower desorption pressure *p*/*p*_0_ and changes in the shape of the desorption branch of the isotherm. The pores of the prepared SBA-15 materials exhibit an average diameter of 70 Å. If part of those pores is narrowed to approximately 50 Å or lower, evaporation is delayed [25–27]. As a result the tensile strength effect is observed at *p*/*p*_0_ = 0.45 leading to a forced closure of the hysteresis loop. Its extent depends on the degree of modification, in particular, the number of narrowed sections created during washing. The height of the bulge in the desorption branch may therefore be used to estimate qualitatively the degree of disorder created by combinational washing with plenty of solvent. However, the forced closure of the hysteresis loop is temperature and adsorptive dependent [30–31]. Therefore argon adsorption/desorption measurements, for example, are a good solution to distinguish more clearly between pores with constrictions, plugs or corrugated surface and to obtain more reliable quantitative information about the pore size distribution [24].

The observed effect on the isotherm shape and the interpretation as blocking or narrowing of the mesopores has also been described by other researchers [25,32–35]. Vansant et al., for instance, reported for Plugged Hexagonal Templated Silica (PHTS), which is a material analogous to SBA-15, synthesized at different TEOS/Pluronic 123 ratios, the occurrence of pore narrowing and blocking by silica nanoparticles inside the pores [25,32–34]. Tian et al. polymerized *N*-isopropylacrylamide inside the SBA-15 structure and explained their XRD results and the isotherm shape with a bulge at *p*/*p*_0_ = 0.45 by the presence of a poly-*N*-isopropylacrylamide layer of varying thickness on the SBA-15 surface [35].

## Conclusion

Washing with plenty of pure solvent (water or ethanol) does not alter the homogeneity and order of the pores in SBA-15. A combined washing approach using water and ethanol increases the surface area, but may change the SBA-15 when plenty of solvent is used. The change can be observed as a bulge at a *p*/*p*_0_ value of 0.45 in the desorption branch of the nitrogen adsorption isotherm (tensile strength effect) and a decrease of the long range order in XRD. This may be attributed to hydrolysis and re-condensation reactions of the silica in the pore wall, which leads to a narrowing or widening of certain pore sections. Thus, controlled washing with reduced quantities of solvent is the optimum condition for obtaining an increased surface area and a narrow monomodal pore size distribution. Scale up of the SBA-15 synthesis reduces the influence of the solvent volume on the shape of the isotherm and the pore size distribution.

## Experimental

### Synthesis of SBA-15

4.07 g Pluronic 123 were dissolved in 30 mL distilled water and 120 mL 2 M hydrochloric acid in a perfluoralkoxylalkane (PFA) bottle at 35 °C. Afterwards, 9 mL of tetraethoxysilane (TEOS) were added and the mixture was stirred (600 rpm) at 35 °C for 20 h in the closed bottle. The resulting white suspension was then aged at 85 °C for 24 h without stirring. After cooling to room temperature, the copolymer was removed by washing with distilled water and/or ethanol using a G4 frit. The product was dried at room temperature over night and then calcined at 550 °C for 12 h. Then the product was carefully ground and characterized by N_2_ adsorption/desorption isotherms. The FWHM and intensity maxima of the pore size distribution were determined by fitting with a Gaussian function. The batches were also increased in size (3×, 9×), as compared to the size described in the literature [1,12]. These batches are referred to as 3× or 9× batches. When they were divided into two smaller batches they were named 3× or 9× half batches. The yield for a 1×, 3× and 9× batch was 2.5 g, 7.5 g and 25 g SBA-15. The residue carbon content of the SBA-15 determined by elemental analysis after calcination was – independent of batch size and washing procedure – 0.09 % at maximum.

### Details of the washing procedure

Two batches produced under the same synthesis conditions can still show small variations regarding surface area, pore volume and pore size distribution, which makes it difficult to study the pure effect of washing. The absolute surface area and pore volume may deviate from batch to batch by ±5%. For the pore size distribution this deviation is about ±2%. To overcome this problem, a batch or scaled up batch was divided into two identical half batches and a small, unwashed reference sample of approximately 80 mg. The weight of the reference samples is negligible compared to the two half batches which are used for the washing experiments. The washing was always performed 15 times with a defined quantity of solvent which was poured on to the sample in the G4 frit at 25 °C and subsequently removed by suction during each washing cycle. Additionally, the sample was carefully stirred with a glass rod after each addition of solvent. The combined washing approach consisted of washing with defined quantities of water (5×), ethanol (5×) and again water (5×). In the case where only pure solvent was used the amount of each solvent quantity and the total number of washing cycles (15×) was comparable as in case of the combined washing approach. In Table 2 typical contact times of the solvents for each washing step with dependence on the batch size are given.

**Table 2:** Typical contact times while washing.

SBA-15 sample	washing procedure^a^	contact time (min)		
		water	ethanol	water

**3A**	no washing	0	0	0
**3B**	5 mL ethanol/water (1× half batch)	2	2	2
**3C**	35 mL ethanol/water (1× half batch)	6	6	6
**3D**	120 mL ethanol/water (3× half batch)	15	10	11
**3E**	350 mL ethanol/water (9× half batch)	60	30	27

^a^ solvent volume is given per washing cycle.

### Nitrogen adsorption

The calcined SBA-15 samples were pre-treated in vacuum at 80 °C for 16 h and then measured on Quantachrome Autosorb-1 and Autosorb-6B instruments. The total pore volume was determined from the adsorption branch of the N_2_ isotherm curve at a relative pressure of *p*/*p*_0_ = 0.95. A standard isotherm was measured with 80 data points. For the calculation of the surface area a nitrogen cross section of 13.5 Å^2^ was used [36]. The pore-size distribution was calculated from the adsorption and desorption branch of the isotherm using the NLDFT method and its FWHM determined by fitting with a Gaussian function. The micro-pore surface area was calculated at *p*/*p*_0_ values from 0.3 to 0.45 with the *t*-plot method by de Boer [37].

### X-ray diffraction

Low angle X-ray diffraction (XRD) measurements were performed on a conventional (i.e., wide angle) STOE STADI P transmission powder diffractometer, equipped with a primary focusing Ge monochromator (Cu Kα_1_ radiation) and scintillation counter. In order to enhance the accuracy of the 2θ scale, a measurement mode with two symmetric scans (negative and positive 2θ) was chosen. Small amounts of powdered sample were sandwiched between two layers of polyacetate film and fixed with a small amount of X-ray amorphous grease. This sandwich was clamped into a sample holder ring, which was rotated around the primary beam axis. At low angles, small differences in 2θ result in significant errors on the d-spacing scale. Thus, the diffractions patterns were evaluated using correlated fitting of the asymmetric diffraction peaks. An asymmetric instrumental function was convoluted with a symmetric Voigt function representing the sample contribution. A common lattice parameter *a* and a common 2θ offset (zero error) was refined on the (100), (110) and (200) peaks of the two-dimensional hexagonal lattice for both scan ranges (negative and positive) simultaneously. Due to the internal 2θ calibration based on the symmetric scan mode and correlated fitting, the instrumental zero error can be determined with high precision, yielding a more reliable determination of the *a*_0_ lattice parameter in turn. Thus, this procedure allows a robust and reproducible evaluation of the d-values of differently treated samples. However, it needs to be kept in mind that, both, due to the asymmetric peak shape and the strongly asymmetric background, these values will depend strongly on the evaluation procedure applied. Thus, care should be taken when comparing the results of different studies on an absolute scale.
